# Gender-related differences in cognitive performance and cognitive stimulation efficacy in subjects with Parkinson’s disease and mild cognitive impairment

**DOI:** 10.3389/fnagi.2025.1672084

**Published:** 2025-10-22

**Authors:** Noemi Iaccino, Jolanda Buonocore, Giusi Torchia, Francesca Curcio, Fabio M. Pirrotta, Marianna Contrada, Loris Pignolo, Antonio Gambardella, Gennarina Arabia

**Affiliations:** ^1^Department of Medical and Surgical Sciences, Institute of Neurology, Magna Graecia University, Catanzaro, Italy; ^2^Neuroscience Research Center, Magna Graecia University, Catanzaro, Italy; ^3^Regional Center for Serious Brain Injuries, S. Anna Institute, Crotone, Italy

**Keywords:** Parkinson’s disease, gender, cognitive reserve, cognitive stimulation, cognitive impairment, neuroscience

## Abstract

**Background:**

Gender-related differences in cognitive performances of subjects with Parkinson’s Disease and mild cognitive impairment (MCI-PD) have been recently investigated in a few studies, yielding heterogeneous results. Cognitive stimulation (CS) is a promising non-pharmacological treatment in MCI-PD subjects and no data regarding gender differences in its efficacy are available yet. The aim of this study was to investigate gender-related differences in cognitive functions and CS efficacy in subjects with MCI-PD.

**Methods:**

Forty-five MCI-PD subjects (30 men, 15 women) were randomized to a 4-week CS program, delivered either via tele-rehabilitation (TR) or with a conventional in-person approach. A broad clinical and neuropsychological assessment, including the Cognitive Reserve Index questionnaire, was conducted at baseline (T1), post-treatment (T2), and at 6-month follow-up (T3).

**Results:**

At baseline, women showed a lower cognitive reserve (CR) compared to men (*p* = 0.039). After adjusting for CR, women performed worse than men in global cognition, attention, and visuospatial abilities. After CS treatment, men demonstrated significant improvements in global cognition, language, executive functions, working memory, visuospatial abilities, attention, and trait anxiety (*p* < 0.05). Women showed significant improvements in global cognition (MoCA, *p* = 0.036) and mood (BDI, *p* = 0.021). Men outperformed women in several domains, both in TR and in-person rehabilitation groups. Regression models revealed a stronger modulatory effect of CR in global cognition, attention, memory, and language, in women. After a 6-month treatment discontinuation, cognitive performance measures significantly worsened in all subjects, regardless of gender.

**Conclusion:**

Our study showed gender-related differences both in cognitive functions and in efficacy of CS in subjects with MCI-PD, also highlighting the role of cognitive reserve. These findings support the relevance of developing gender-tailored cognitive rehabilitation strategies to enhance treatment outcomes and improve the quality of life for individuals with MCI-PD.

## 1 Introduction

Mild cognitive impairment (MCI) is a common non-motor manifestation of Parkinson’s disease (PD), affecting approximately 27% of subjects without dementia (Litvan et al., 2011) Referred to as MCI-PD, this condition often presents with impairments in executive functions, attention, visuospatial skills, and verbal fluency, frequently associated with slowed thinking (bradyphrenia). In many cases, the clinical picture is further complicated by the coexistence of some neuropsychiatric symptoms, such as depression, anxiety, and apathy (Litvan et al., 2012; Biundo et al., 2016).

Some studies have explored potential gender differences in cognitive functioning among individuals with PD, though findings remain heterogeneous. Some evidence suggests that men may be more vulnerable to decline in memory (Liu et al., 2015), verbal fluency (Locascio et al., 2003; Reekes et al., 2020; Oltra et al., 2022), and executive functions (Reekes et al., 2020), while women may show greater difficulties in visuospatial processing (Locascio et al., 2003; Liu et al., 2015; Oltra et al., 2022) Additionally, male sex has been proposed as a risk factor for cognitive decline and the development of dementia in PD (Cereda et al., 2016). Additionally, men with PD presenting with an akinetic-rigid phenotype (Cerri et al., 2019) or carrying GBA mutations (Phongpreecha et al., 2020) exhibited a higher risk of developing cognitive impairment. These observations highlighted that gender may influence not only baseline cognitive profiles but also the trajectory of cognitive changes over time.

Cognitive reserve, i.e., the capacity of the brain to compensate for pathology through pre-existing cognitive resources built up through education, occupation, and other life experiences (Stern et al., 2020), may act as a modulating factor for cognitive performance. Indeed, a higher cognitive reserve has been linked to better cognitive functioning and slower decline in various neurodegenerative conditions, including PD (Stern, 2012; Hindle et al., 2016), even though how it interacts with gender in MCI-PD remains unknown.

While new disease-modifying pharmacological options for cognitive impairment in PD are under investigation, interest has grown towards non-pharmacological interventions, such as cognitive rehabilitation (Goldman and Sieg, 2020). Cognitive stimulation (CS) is one of the most widely used approaches. Previous studies have suggested potential benefits of CS for subjects with MCI-PD (Folkerts et al., 2018), although existing research data are often limited by small sample sizes, heterogenous methodologies, and short follow-up durations. Tele-rehabilitation (TR) has emerged more recently as a potentially viable and accessible alternative to traditional in-person approaches, particularly for individuals with mobility or logistical constraints (Cacciante et al., 2022). To date, no data on gender influences on response to cognitive rehabilitation in MCI-PD are available.

This study was aimed at investigating gender differences in subjects with MCI-PD: (a) in baseline cognitive performances, evaluated through a broad structured neuropsychological test battery; (b) in the efficacy of CS performed, either in TR or in a conventional face-to-face setting.

## 2 Materials and methods

### 2.1 Participants and eligibility criteria

A total of 45 participants (30 men and 15 women) with MCI-PD were consecutively recruited, between January 2022 and June 2024, at the Neurology Unit of the “Magna Graecia” University Hospital in Catanzaro, Italy. Participants were eligible if they fulfilled the clinical diagnostic criteria for MCI-PD, according to Movement Disorder Society -Level I criteria (Litvan et al., 2012). Cognitive impairment was identified on the basis of a limited neuropsychological battery (i.e., the battery includes less than two tests within each of the five cognitive domains, or less than five cognitive domains are assessed) with a performance two standard deviations below age- and education-adjusted Italian normative data on a validated global cognitive screening tool for PD, such as Montreal Cognitive Assessment, MoCA (Nasreddine et al., 2005), or on at least two neuropsychological tests, with preservation of functional independence as determined by Activities of Daily Living (ADL) and Instrumental Activities of Daily Living (IADL) scores. Only individuals aged between 50 and 85 years and on stable pharmacological treatment, for at least 4 weeks prior to baseline, were included. Subjects were excluded if they had: (a) other neurological diseases likely to affect cognition (e.g., epilepsy, multiple sclerosis, moderate-to-severe traumatic brain injury), (b) a current major psychiatric disorder (e.g., psychotic disorders or severe depression with active symptoms), (c) significant uncorrected sensory or language impairments, or (d) any medical condition that might compromise compliance with study procedures. Among the 123 eligible subjects, 78 were excluded because they either did not meet the inclusion/exclusion criteria or refused to participate. More in details, 52 subjects did not meet eligibility criteria, 16 refused participation because of technology barriers (9 men and 7 women), and 10 refused without a reported reason (5 men and 5 women). Thus, the enrolment rate was 38.96% for men and 31.91% for women.

The study was conducted in accordance with the Declaration of Helsinki and approved by the Regional Ethics Committee. All participants gave written informed consent prior to enrollment. This work was carried out within the framework of the MULTIPLAT_AGE national research project (Pilotto et al., 2024), which promotes integrated care strategies for older adults with multimorbidity in Italy.

### 2.2 Clinical assessment

All participants underwent a comprehensive neurological and neuropsychological evaluation. Neurological assessment included the Disorder Society - Unified Parkinson’s Disease Rating Scale (MDS-UPDRS) (Goetz et al., 2008) and the Hoehn and Yahr Scale (H-Y) (Goetz et al., 2004). Clinical assessment also included Mini Nutritional Assessment (MNA) (Vellas et al., 1999), Cumulative Illness Rating Scale (CIRS) (Linn et al., 1968), Activities of Daily Living (ADL) (Devi, 2018), Instrumental Activities of Daily Living (IADL) (Mathuranath et al., 2005). Multidimensional Prognostic Index (MPI) (Dent et al., 2016), Caregiver Burden Inventory (CBI) (Novak and Guest, 1989) and 36-Item Short Form Survey (SF-36) were used for evaluation of caregiver burden and quality of life (Ware and Sherbourne, 1992). The Levodopa Equivalent Daily Dose (LEDD) (Tomlinson et al., 2010) was calculated to quantify the total daily intake of antiparkinsonian drugs. In the neuropsychological evaluation, global cognition [Clinical Dementia Rating, CDR (Morris, 1993), Mini-Mental State Examination, MMSE (Folstein et al., 1975), Montreal Cognitive Assessment, MoCA (Nasreddine et al., 2005)] was assessed along with verbal memory [Rey Auditory Verbal Learning Test—immediate and delayed recall (Ricci et al., 2022)], visuospatial abilities [Copy Drawing CD/CDP (Gainotti et al., 1977)], short term and working memory [Digit Span Forward, FW and Backward, BW (Monaco et al., 2013)], attention [Trail Making Test A/B, TMT-A/B (Giovagnoli et al., 1996)] and executive functions (Frontal Assessment Battery, FAB [Dubois et al., 2000)]. Language was assessed using phonemic fluency [FAS (Carlesimo et al., 1996)] and Battery for the Analysis of Aphasic Deficits [BADA (Miceli et al., 1994) names and verbs] for naming and verb generation. Beck Depression Inventory-2 [BDI-2 (Richter et al., 1998)] and State–Trait Anxiety Inventory (STAI X-1/STAI X-2) were used to evaluate emotional status (Spielberger, 1966). Cognitive reserve was evaluated using the Cognitive Reserve Index questionnaire (CRIq). The CRIq questionnaire assesses an individual’s cognitive reserve by gathering information about his/her entire adult life. It is divided into three sections: CRI-School, CRI-Work, and CRI-FreeTime (Nucci et al., 2019).

The assessments were conducted at baseline, before starting any interventions (T1), after 20 sessions of CS (T2), and at a follow-up time, 6 months after the end of the CS treatment (T3). To limit test adaptation or learning effects between T1 and T2 evaluations, parallel versions of the scales, or item rotation within them, were applied, when possible.

### 2.3 Cognitive intervention

All participants underwent a structured four-week CS program consisting of 20 sessions (five sessions per week, each lasting approximately 45 min). CS was delivered either through a tele-rehabilitation (TR) system (tele-rehabilitation group, TRG) or with a traditional face-to-face program (control group, CG). An independent researcher (L.P.), not involved in recruitment or treatment delivery, generated the block randomization sequence, using a computer-based procedure. Because of the type of interventions, blinding of participants and therapists was not feasible. However, outcome assessment was kept blind. Baseline, post-intervention, and follow-up evaluations were carried out by neuropsychologists unaware of group allocation. Participants were instructed not to reveal their assignment during testing. The CS program was standardized across the two groups in terms of duration, structure, and domains targeted and promoted general cognitive engagement. Participants in the TRG performed synchronous CS, using the VRRS-HomeKit (Khymeia, Italy), a digital platform enabling remote interaction between subjects with PD-MCI and the therapists. Sessions were conducted live, allowing real-time adjustments to task difficulty and pacing, and focused on general cognitive activation across domains including attention, executive functioning, memory, and visuospatial abilities. The CG received traditional face-to-face cognitive stimulation in outpatient clinics, administered by trained neuropsychologists. Sessions included paper-and-pencil exercises matched in cognitive domain and complexity to those delivered in the TR group, ensuring methodological equivalence between conditions. Materials included validated clinical exercises commonly used in neuropsychological practice, ensuring methodological equivalence across delivery formats. Activities were adapted to maintain engagement, but no hierarchical progression was applied. This approach guaranteed comparable cognitive stimulation between groups, differing only in the modality of delivery.

### 2.4 Statistical analysis

All statistical analyses were performed using R (version 2025.05.0 + 496). Descriptive statistics were used to summarize clinical and demographic characteristics. Continuous variables expressed as means and standard deviations (SD). Baseline (T1) comparisons between men and women were performed using independent-samples t-tests for normal distribution or the Mann–Whitney U test for non-normal distribution, depending on the results of the Shapiro–Wilk normality test. The Wilcoxon test was used for comparisons between measurements over time. We also conducted a Multivariate Analysis of Covariance (MANCOVA), including any significant variable at baseline as a covariate. Separate multiple linear regression models were used to explore if the treatment effect and the influence of significant variables at baseline on cognitive outcomes were modulated by gender. To account for multiple comparisons, all *p*-values were adjusted for a false discovery rate (FDR) of 0.05 using the Benjamini Hochberg method.

## 3 Results

### 3.1 Sample characteristics and gender-based differences at baseline

At baseline (T1), there were no significant between-group differences (men vs. women) in age, disease duration, duration of cognitive symptoms, years of education, or LEDD. Functional independence, mood, and comorbidity burden were also comparable between men and women (Table 1). A significant difference emerged for cognitive reserve, with men having higher scores of CRIq in comparison to women (*p* = 0.039). A trend of lower scores in the other neuropsychological tests was observed in women compared to men, even though this did not reach the statistical threshold. Using MANCOVA test with the CRIq as a covariate, significant differences were found in MoCA (*p* = 0.017, *F* = 6.96), Copy of Drawings – CD (*p* = 0.031, *F* = 5.51), and TMT-A (*p* = 0.045, *F* = 4.69), indicating poorer performances in global cognition, visuospatial abilities and attention, respectively, in women compared to men. Furthermore, women also exhibited a higher fragility index (*p* = 0.013, *F* = 6.82).

**Table 1:** Baseline demographic and clinical characteristics of men and women participants.

Data	Men (*n* = 30)	Women (*n* = 15)	*p*-value
Age (years)	70.70 ± 6.57	70.87 ± 7.88	0.92
PD duration (years)	6.76 ± 4.08	6.40 ± 4.22	0.77
MCI duration (years)	1.90 ± 1.36	1.77 ± 1.21	0.75
Education (years)	9.73 ± 3.44	8.40 ± 4.85	0.15
LEDD	384.14 ± 202.32	437.00 ± 216.05	0.43
CRIq	100.50 ± 20.87	87.67 ± 18.95	**0.039**
H-Y	2.11 ± 0.50	2.07 ± 0.47	0.83
MDS-UPDRS I	12.63 ± 6.42	11.93 ± 6.68	0.92
MDS-UPDRS II	11.67 ± 7.93	12.57 ± 8.37	0.65
MDS-UPDRS III	26.36 ± 12.75	28.43 ± 13.92	0.69
MDS-UPDRS IV	1.93 ± 4.04	1.93 ± 3.25	0.79
CBI	16.37 ± 18.42	16.33 ± 12.60	0.55
Barthel	2.82 ± 0.48	2.36 ± 1.15	0.20
MNA	11.54 ± 2.53	10.14 ± 3.25	0.18
CIRS	3.93 ± 2.00	4.57 ± 2.28	0.26
ADL	5.54 ± 0.69	4.86 ± 1.70	0.58
IADL	5.21 ± 1.83	5.71 ± 2.09	0.39
MPI	0.28 ± 0.09	0.39 ± 0.20	0.075
SF36 tot	1899.14 ± 583.95	1639.86 ± 874.93	0.27

Statistical comparisons were performed using Mann–Whitney U test. All p-values were adjusted to control for FDR using the Benjamini-Hochberg procedure. PD, Parkinson’s disease; MCI, Mild cognitive impairment; LEDD, Levodopa equivalent daily dose; CRIq, Cognitive Reserve Index questionnaire total score; H-Y, Hoehn and Yahr scale; MDS-UPDRS, Movement Disorder Society-Unified Parkinson’s Disease Rating Scale; CDR, Clinical Dementia Rating; STAIX1, State Anxiety Inventory; STAIX2, Trait Anxiety Inventory; CBI, Caregiver Burden Inventory; MNA, Mini Nutritional Assessment; CIRS, Cumulative Illness Rating Scale; ADL, Activities of Daily Living; IADL, Instrumental Activities of Daily Living; MPI, Multidimensional Prognostic Index; SF-36, 36-Item Short Form Survey. Significant *p* values are highlighted in bold.

### 3.2 Longitudinal changes by gender and treatment

**Figure 1:**
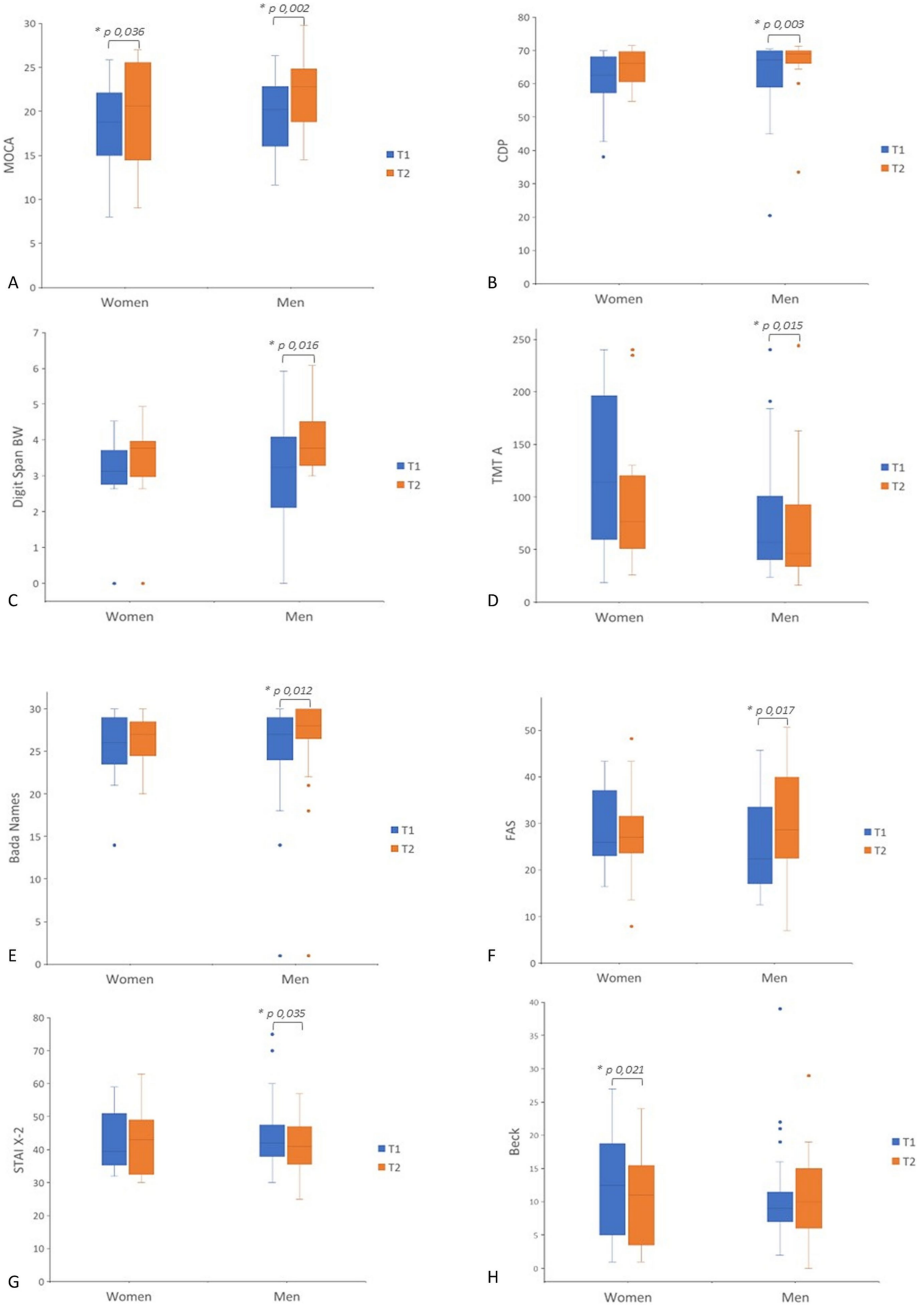
Box plot of pre- to post-treatment scores (T1–T2) in men and women. Separate panels show scores for each outcome measure. **(A)** MoCA; **(B)** CDP; **(C)** Digit Span BW; **(D)** TMT-A; **(E)** BADA Names; **(F)** FAS; **(G)** STAI X-2; **(H)** Beck. Statistical significance was considered for *p* < 0.05. MOCA, Montreal Cognitive Assessment; CDP, Copy Drawing Performance; BW, Digit Span Backward; TMTA, Trail Making Test Part A; T BADA, Battery for the Analysis of Aphasic Deficits; FAS, Phonemic Verbal Fluency; Beck; STAIX2, Trait Anxiety Inventory.

In men, significant improvement in several cognitive domains after CS treatment was observed (Table 2; Figure 1). MoCA scores notably increased (*p* = 0.002), reflecting an enhancement in global cognition. Improvements were also observed in language and executive functions, as indicated by the FAS test (*p* = 0.017), and the BADA Naming test names (*p* = 0.012). Significant improvements were observed in both Digit Span Backward (*p* = 0.016) and Digit Span Forward (*p* = 0.026), indicating enhanced working memory performances. Additionally, visuospatial abilities improved significantly, as shown by the Copy of Drawings CDP test (*p* = 0.003). Processing speed and attention improved, as evidenced by better performance in TMT-A (*p* = 0.015). Men also exhibited a reduction in trait anxiety, as evidenced by a decrease in their STAI X-2 scores (*p* = 0.035).

**Table 2:** Neuropsychological test scores by gender and timepoint (T1, T2).

Data	Men			Women		
	T1	T2	*p*-value	T1	T2	*p*-value
MMSE	25.14 ± 3.60	25.39 ± 3.70	0.77	23.57 ± 3.33	24.93 ± 3.81	0.068
MOCA	19.66 ± 4.21	21.99 ± 3.73	**0.002**	18.07 ± 5.16	19.80 ± 6.04	**0.036**
Rey immediate	33.76 ± 7.87	33.55 ± 9.84	0.80	36.55 ± 10.23	38.33 ± 12.58	0.38
Rey delayed	7.08 ± 3.11	7.20 ± 3.08	0.52	8.01 ± 4.50	7.86 ± 4.65	0.67
CD	8.66 ± 2.59	8.96 ± 2.70	0.39	8.54 ± 3.03	8.81 ± 3.06	0.61
CDP	62.67 ± 10.75	66.71 ± 7.24	**0.003**	60.36 ± 9.90	65.11 ± 4.95	0.07
Digit Span FW	4.66 ± 0.92	5.09 ± 0.80	**0.026**	4.94 ± 1.04	5.17 ± 1.11	0.35
Digit Span BW	2.91 ± 1.78	3.99 ± 0.84	**0.016**	2.76 ± 1.51	3.43 ± 1.11	0.22
TMTA	85.58 ± 64.33	69.39 ± 59.88	**0.015**	125.39 ± 83.29	102.43 ± 76.77	0.17
TMTB	228.92 ± 155.76	204.58 ± 151.73	0.09	327.32 ± 147.64	289.27 ± 140.13	0.08
BADA Nouns	26.31 ± 3.97	27.52 ± 3.15	**0.012**	25.33 ± 4.25	26.60 ± 3.00	0.16
BADA Verbs	23.45 ± 4.08	24.63 ± 3.73	0.06	21.40 ± 5.44	22.13 ± 4.98	0.27
FAB	13.54 ± 3.24	14.73 ± 2.92	0.15	13.08 ± 3.10	13.79 ± 3.07	0.37
FAS	24.92 ± 9.54	30.13 ± 11.79	**0.017**	28.35 ± 8.51	27.68 ± 10.91	0.61
Beck	11.04 ± 7.59	12.64 ± 9.02	0.59	12.7 ± 9.02	9.7 ± 7.04	**0.021**
STAIX1	42.25 ± 9.19	40.71 ± 9.14	0.41	40.7 ± 8.8	42.0 ± 9.7	0.84
STAIX2	44.71 ± 10.44	43.57 ± 9.90	**0.035**	43.5 ± 9.6	41.7 ± 10.1	0.26

Values are presented as mean ± standard deviation. Statistical comparisons were performed using Wilcoxon Test. All p-values were adjusted to control for FDR using the Benjamini-Hochberg procedure. MMSE, Mini-Mental State Examination; MOCA, Montreal Cognitive Assessment; CD, Copy Drawing -CD; CDP, Copy Drawing Performance; FW, Digit Span Forward; BW, Digit Span Backward; TMTA, Trail Making Test Part A; TMTB, Trail Making Test Part B; BADA, Battery for the Analysis of Aphasic Deficits; FAB, Frontal Assessment Battery; FAS, Phonemic Verbal Fluency; Beck; STAIX1, State Anxiety Inventory; STAIX2, Trait Anxiety Inventory. Significant *p* values are highlighted in bold.

In women, the MoCA scores significantly increased between T1 and T2 (*p* = 0.036), reflecting global cognition enhanced by CS. Moreover, the BDI also demonstrated a significant improvement in mood after intervention (*p* = 0.021).

#### 3.2.1 Gender-related outcomes in TRG and CG

Among the 45 participants who underwent the CS, 25 subjects (15 men and 10 women) performed tele-rehabilitation, and 20 subjects (15 men and 5 women) received face-to-face rehabilitation.

In the TRG, men demonstrated significantly greater improvement in attentive functions after CS (TMT-A, *p* = 0.006; TMT-B, *p* = 0.045). In terms of language skills, all genders showed improvement, after treatment, in the BADA Naming Task, reaching statistically significant results only in men (Names, *p* = 0.013; Verbs, *p* = 0.043). No statistically significant improvements were observed in women in the TRG.

In the control group, after cognitive stimulation, men outperformed women in several areas. They exhibited higher scores on the MoCA (*p* = 0.014), better visuospatial abilities as measured by the Copy of Drawing Test (*p* = 0.025), improved working memory on the Digit Span BW test (*p* = 0.045), greater verbal fluency in the FAS task (*p* = 0.030), and lower anxiety levels according to the STAI-X2 (*p* = 0.027). Among women in the CG, a significant improvement was observed only in the BADA Naming Task for verbs (*p* = 0.048) (Tables 3–4).

**Table 3:** Average of the scores obtained in men and women of TRG in T1 and T2 evaluation.

Data	T1	T2	*p* value
TMTA Men	75.0 ± 62.2	58.0 ± 57.0	**0.006**
TMTA Women	137.3 ± 98.0	109.3 ± 92.7	0.40
TMTB Men	211.2 ± 140.0	176.2 ± 126.1	**0.045**
TMTB Women	293.0 ± 169.4	280.7 ± 154.6	0.59
BADA names Men	23.8 ± 7.9	25.2 ± 7.9	**0.013**
BADA names Women	24.4 ± 4.8	26.0 ± 3.3	0.18
BADA verbs Men	23.7 ± 4.1	24.6 ± 4.6	**0.043**
BADA verbs Women	20.8 ± 6.3	21.3 ± 5.7	0.83

Values are presented as mean ± standard deviation. All p-values were adjusted to control for FDR using the Benjamini-Hochberg procedure. Abbreviations: TMTA, Trail Making Test Part A; TMTB, Trail Making Test Part B; BADA, Battery for the Analysis of Aphasic Deficits. Significant *p* values are highlighted in bold.

**Table 4:** Average of the scores obtained in men and women of CG in T1 and T2 evaluation.

Data	T1	T2	*p* value
MoCA Men	19.4 ± 4.1	21.9 ± 4.1	**0.014**
MoCA Women	22.0 ± 2.69	24.9 ± 2.3	0.06
CDP Men	61.7 ± 14.0	66.6 ± 9.7	**0.025**
CDP Women	65.1 ± 4.2	67.8 ± 4.0	0.44
BW Men	3.1 ± 2.0	4.4 ± 0.9	**0.045**
BW Women	2.7 ± 1.9	4.2 ± 0.6	0.13
FAS Men	25.3 ± 10.4	30.7 ± 9.9	**0.030**
FAS Women	34.8 ± 9.3	36.16 ± 10.8	0.71
STAI X-2 Men	40.7 ± 6.7	37.9 ± 6.7	**0.027**
STAI X-2 Women	37.2 ± 5.9	34.0 ± 6.4	0.14
BADA verbs Men	23.9 ± 3.9	24.6 ± 2.9	0.40
BADA verbs Women	22.6 ± 3.3	23.8 ± 2.9	**0.048**

Values are presented as mean ± standard deviation. All p-values were adjusted to control for FDR using the Benjamini-Hochberg procedure. MOCA, Montreal Cognitive Assessment; CDP, Copy Drawing Performance; BW, Digit Span Backward; FAS, Phonemic Verbal Fluency; STAIX2, Trait Anxiety Inventory; BADA, Battery for the Analysis of Aphasic Deficits. Significant *p* values are highlighted in bold.

We performed a repeated-measures ANOVA to compare pre- and post-treatment values (T1 vs. T2) and to test the interaction between treatment modality (tele-rehabilitation vs. face-to-face) and time in both men and women, examining the general outcomes. No statistically significant differences were observed in any performed comparison (Supplementary Table S1).

#### 3.2.2 Follow- up at 6 months

In men and women, cognitive and functional measures showed a worsening after 6-month treatment discontinuation (Table 5). Men presented a significantly greater decline in visuospatial domain (Copy of Drawings CDP: *p* = 0.014), in short term and working memory (Digit Span FW: *p* = 0.0290, Digit Span BW: *p* = 0.014), and verbal fluency (BADA Names: *p* = 0.0015, BADA Verbs: *p* = 0.0008), comparing T2 and T3 time-point evaluations.

**Table 5:** Neuropsychological assessment at T2 and T3 evaluation in men and women.

Data	**Men**			**Women**		
	T2	T3	*p* value	T2	T3	*p* value
MOCA	21.9 ± 3.5	20.8 ± 4.5	**0.0160**	19.9 ± 5.8	19.8 ± 5.2	0.99
Rey immediate	33.6 ± 9.1	32.7 ± 9.1	0.58	38.06 ± 12.2	36.3 ± 12.1	0.43
Rey delayed	7.2 ± 2.9	6.1 ± 2.9	0.048	7.8 ± 4.5	6.7 ± 4.0	0.13
CD	9.0 ± 2.6	8.5 ± 2.7	0.14	8.8 ± 3.1	8.7 ± 3.2	0.56
CDP	66.9 ± 6.9	62.5 ± 13.7	**0.014**	65.1 ± 4.9	62.8 ± 7.6	0.34
Digit Span FW	5.1 ± 0.8	4.8 ± 0.8	**0.029**	5.2 ± 1.1	4.3 ± 1.1	0.14
Digit Span BW	3.9 ± 0.8	3.4 ± 1.5	**0.014**	3.4 ± 1.1	2.6 ± 1.9	0.29
TMTA	68.7 ± 57.8	84.5 ± 74.2	0.09	102.4 ± 76.8	115.2 ± 78.9	0.89
TMTB	201.7 ± 143.9	247.2 ± 146.4	**0.0029**	289.3 ± 140.1	310.2 ± 142.8	0.50
BADA Nouns	27.6 ± 2.9	25.96 ± 4.7	**0.0015**	26.6 ± 3.0	26.4 ± 2.43	0.93
BADA Verbs	24.7 ± 3.5	21.9 ± 5.6	**0.0008**	22.1 ± 5.0	20.9 ± 5.0	0.16
FAB	14.8 ± 2.7	14.2 ± 3.3	0.52	13.9 ± 3.0	13.7 ± 2.7	0.75
FAS	29.8 ± 10.9	27.5 ± 8.2	0.64	27.7 ± 10.5	26.8 ± 7.0	0.64
Beck	10.2 ± 6.1	12.2 ± 6.4	0.12	10.7 ± 7.7	14.2 ± 10.2	0.04
STAIX1	40.7 ± 8.3	42.2 ± 9.3	0.39	42.0 ± 9.7	42.2 ± 13.5	0.98
STAIX2	41.1 ± 6.9	40.2 ± 8.3	0.68	41.7 ± 10.1	41.8 ± 10.5	0.91

Values are presented as mean ± standard deviation. All p-values were adjusted to control for FDR using the Benjamini-Hochberg procedure. MOCA, Montreal Cognitive Assessment; CD, Copy Drawing -CD; CDP, Copy Drawing Performance; FW, Digit Span Forward; BW, Digit Span Backward; TMTA, Trail Making Test Part A; TMTB, Trail Making Test Part B; BADA, Battery for the Analysis of Aphasic Deficits; FAB, Frontal Assessment Battery; FAS, Phonemic Verbal Fluency; Beck; STAIX1, State Anxiety Inventory; STAIX2, Trait Anxiety Inventory. Significant *p* values are highlighted in bold.

### 3.3 Interaction between CRIq scores and gender on cognitive outcomes

**Figure 2:**
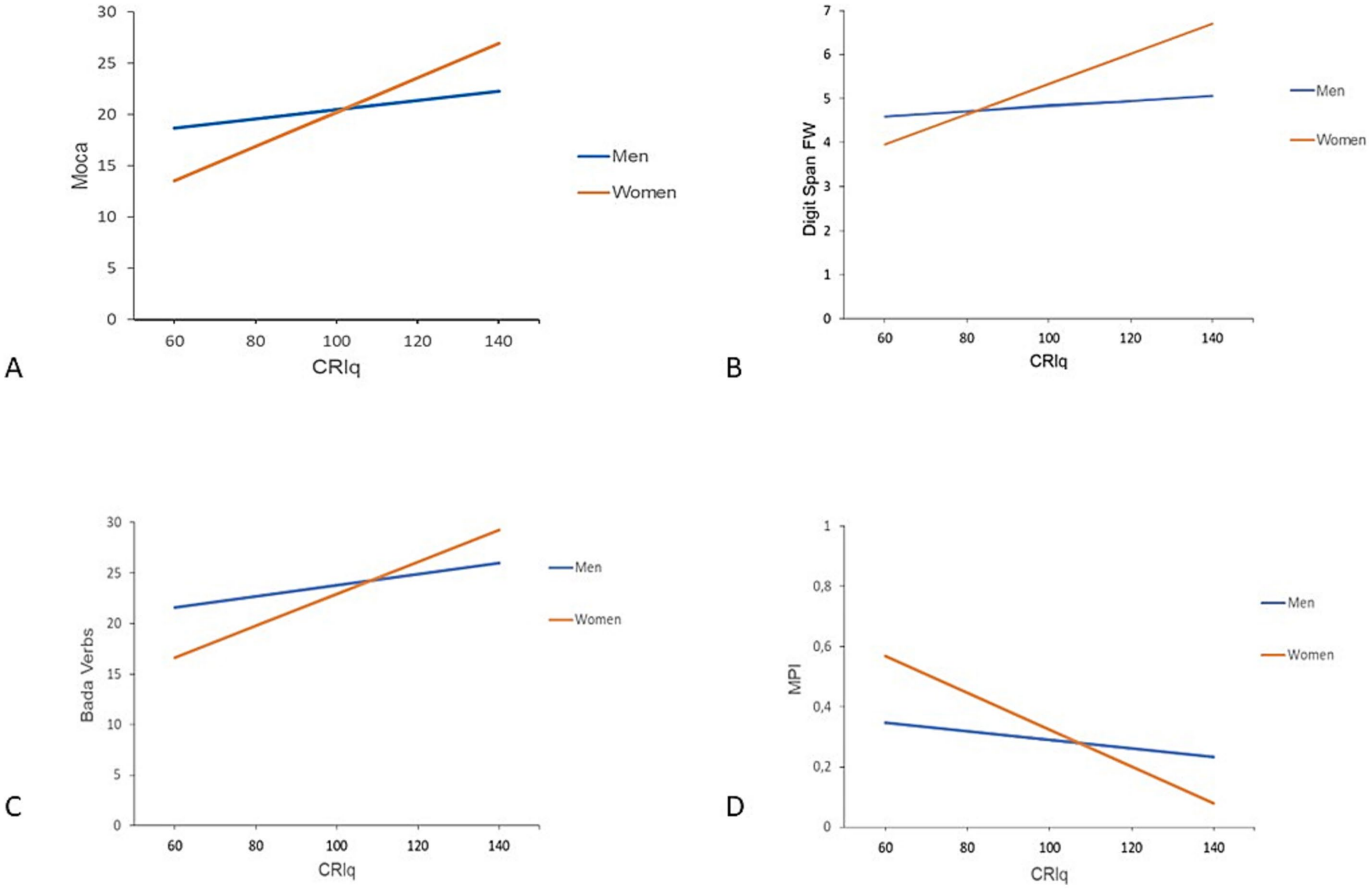
Interaction effect between CRIq and gender on cognitive outcomes. The plot shows the regression lines for men (blue) and women (orange), illustrating the interaction between CRIq and gender on cognitive outcomes. **(A)** MoCA; **(B)** Digit Span—FW; **(C)** BADA Verbs; **(D)** MPI. MOCA, Montreal Cognitive Assessment; FW, Digit Span Forward; BADA, Battery for the Analysis of Aphasic Deficits; MPI, Multidimensional Prognostic Index.

In the linear regression model for MoCA, the CRIq × gender interaction was significant (β = 0.119; *p* = 0.023), with the predictors accounting for 25% of the variance (R^2^ = 0.250), suggesting that the effect of CRIq on MoCA scores was stronger in women than men. Similarly, for Digit Span Forward, the model explained 19.1% of the variance (R^2^ = 0.191), with a significant CRIq × gender interaction (β = 0.028; *p* = 0.008). For BADA Verbs, the model was also significant (R^2^ = 0.252), with a significant interaction effect (β = 0.104; *p* = 0.035), again showing a stronger association between CRIq and performance in women. Finally, in the model for MPI, higher CRIq scores predicted a reduction in frailty indices, with this effect being more pronounced in women than in men (β = 0.0046; *p* = 0.002, R^2^ = 0.352). Figure 2 shows the interaction effects between CRIq and gender on cognitive outcomes for MoCA, Digit Span Forward, BADA Verbs, and MPI scores.

## 4 Discussion

To our knowledge, this is the first study investigating how gender may modulate cognitive performance and rehabilitation efficacy in individuals with MCI-PD. We found that, at baseline, women exhibited greater vulnerability in several cognitive domains, including global cognition, attention, and visuospatial processing, after adjusting for cognitive reserve. Indeed, in our cohort, women showed a lower cognitive reserve compared to men, even though they had comparable age, education, and sociographic background. This finding highlighted the differential interactions between gender and cognitive reserve in shaping cognitive functions.

Our findings have relevant clinical implications. Gender differences in PD are increasingly recognized, some studies report different patterns in epidemiology, disease risk factors, clinical presentation, and treatment response between men and women. Other insights into these sex- and gender-specific differences could lead to improvements in the diagnosis and treatment of PD. Clinical trials should include sex and gender as key factors in their analyses. As a result, medical guidelines should be updated to reflect these differences, enhancing the quality and suitability of care for all patients. Characterizing gender-related differences in PD with cognitive impairment could be useful for developing personalized therapy from the early stages of disease and setting a treatment as targeted as possible.

Most studies assessing gender differences in cognitive performance have focused on *de novo* PD population. According to our results, those studies have reported that women showed a greater impairment in visuospatial abilities (Locascio et al., 2003; Oltra et al., 2022). They also suggested that men may be more susceptible to declines in memory, verbal fluency, and executive functions. Bayram et al. (2020) includes both de novo PD and PD-MCI participants. Their findings confirmed the poorer visuospatial performance in women, along with better verbal memory performance compared to men. However, heterogeneity in methodological approaches of the previous studies, including adjustment for cognitive reserve, may have influenced the outcomes. The role of cognitive reserve has been more extensively investigated in Alzheimer’s Disease (AD), where women have been shown to have lower cognitive reserve and greater vulnerability to cognitive decline (Kim et al., 2024). A 2024 narrative review further explored how men and women showed different profiles of resilience and resistance to pathological biomarkers, brain atrophy, and cognitive decline: women tend to maintain memory function more effectively in the early stages, but highlighted a marked decline with the progression of the disease (Arenaza-Urquijo et al., 2024). Recent studies have also evaluated it in the context of PD (Hindle et al., 2016; Ophey et al., 2024). A meta-analysis performed by Gu et al. confirmed that elevated levels of cognitive reserve are associated with a reduced risk of cognitive decline in PD subjects (Gu and Xu, 2022). One possible explanation for gender-related differences in cognitive reserve could lie in early-life experiences, including sociocultural and educational disparities. As suggested by Jones et al., men and women may be exposed to different educational expectations and learning environments, which may influence cognitive development over time (Jones and Wheatley, 1990).

In our study, women also showed a higher frailty index in comparison to men, using the MPI assessment. This result is consistent with previous researches indicating a greater prevalence of frailty among older women, even without PD. Indeed, several studies have demonstrated that women tend to accumulate more health deficits with age, leading to higher frailty scores compared to men (Roland et al., 2012; Gordon et al., 2017).

Our study revealed gender-specific patterns in the response to rehabilitative treatment, with greater cognitive improvement in men in comparison to women.

In our sample, men exhibited lower cognitive impairment at baseline, however, they demonstrated greater improvement following treatment compared to women. More in detail, in men, the most notable gains were found in global cognition, executive functions, working memory, attention, visuospatial abilities, language, and anxiety, while women showed marked enhancements in global cognition and mood. According to our research, previous data suggesting that CS was more effective when administered in the early stages of cognitive decline (Clare and Woods, 2004; Giustiniani et al., 2022). Notably, men appeared to benefit more extensively from both interventions. In the TRG, men exhibited significant improvements in attention and verbal fluency, whereas women in the same group did not demonstrate significant changes. In the CG, men also outperformed women in global cognition, visuospatial abilities, and working memory, indicating broad cognitive benefits. Additionally, men of CG also showed improvements in verbal fluency and a significant reduction in anxiety levels, highlighting the potential affective benefits of structured in-person engagement. For women in the control group, improvement was limited to the verb-naming task. This supports the hypothesis that both face-to-face and remote interventions can contribute to the improvement of cognitive function in PD, as previously noted in the TR literature (Maggio et al., 2024). A meta-analysis by Giustiniani et al. evaluated the efficacy of cognitive rehabilitation in MCI-PD and found domain-specific improvements, particularly in global cognition, executive functioning, and both long- and short-term memory, but not in attention and visuospatial domains (Giustiniani et al., 2022). Another systematic review and meta-analysis, which included 12 studies with 512 participants, found moderate improvements in global cognition and working memory, as well as small but significant improvements in verbal memory and executive function (Sanchez-Luengos et al., 2021). These findings underscore the importance of tailoring interventions to the specific cognitive deficits presented by each patient, as not all domains may respond equally to treatment. Importantly, our findings indicate that both TR and traditional face-to-face interventions can effectively improve cognitive functions in PD, particularly in areas typically affected by the disease (Cornejo Thumm et al., 2021; Bianchini et al., 2022).

Our results support the role of cognitive reserve also in the differential effectiveness of CS in men and women. The impact of cognitive reserve appears more pronounced in women than in men, especially in some domains, such as attention and short-term memory, language processing, and episodic memory, suggesting a gender-modulated mechanism of cognitive reserve. Most studies linking cognitive reserve to outcomes in cognitive stimulation interventions have been conducted in AD, where women experienced greater benefits from CR in delaying the clinical onset of the disease (Chooi et al., 2022). In PD, one study investigating the impact of cognitive reserve on the effectiveness of balance rehabilitation found that individuals with lower cognitive reserve showed greater improvements (Piccinini et al., 2018). Carbone et al. highlighted the role of CR in shaping both short- and long-term cognitive and psychological outcomes following CS therapy in individuals with mild to moderate dementia. Their results support the idea that higher levels of cognitive reserve are associated with greater treatment benefits, which aligns with our observation that CRIq scores significantly modulate cognitive improvement, especially in women.

At the six-month follow-up, global cognitive performance declined in all subjects, with specific patterns in some domains. In particular, a more significant decline was observed in the visuospatial domain, short-term and working memory, and verbal fluency, in men. These results suggest that while CS yields measurable short-term benefits, ongoing stimulation may be required to maintain these gains over time, particularly in domains more vulnerable to decline. Taken together, these results confirm the potential of cognitive stimulation in MCI-PD to promote improvement across cognitive domains. The emergence of domain-specific patterns suggests that benefits are not uniformly distributed, but rather shaped by individual characteristics, such as gender and cognitive reserve. These findings resonate with current models of personalized neurorehabilitation, which advocate tailoring interventions based on demographic, clinical, and cognitive profiles (Sousa et al., 2021).

Our study has some limitations. The relatively small sample size, especially within gender-treatment subgroups, may have reduced the power to detect complex interactions and may account for the borderline significance of some results. Moreover, the efficacy of CS in MCI-PD is not yet firmly established; thus, our findings should be considered exploratory and interpreted with caution. Nonetheless, consistent trends observed across analytical methods lend robustness to the conclusions. Furthermore, the number of women with MCI-PD who were willing to participate to our rehabilitation study was lower than that of men, in particular in the face-to-face group. This data is consistent with the evidence coming from previous studies, which have highlighted the greater challenges often encountered in engaging women in such interventions (Willis et al., 2011). Women are less likely to receive specialized care than men with access to treatments often delayed. Moreover, women have less probability than men to get support from informal caregivers, such as their family members (Dahodwala et al., 2018). Lastly, the 6-month follow-up period could have limited longitudinal data, but the selected three timepoints for assessment captured a relevant window for evaluating short-term change around the rehabilitative intervention and the following temporal trajectory of the cognitive functions. Indeed, after cognitive improvement due to the CS treatment, at the 6-month evaluation most of the neuropsychological scores reverted to the baseline values.

Consideration should be given to implementing CS programs for both men and women, as both the TR program and face-to-face interventions have been reported to be effective. The main benefit of TR is enhanced accessibility, especially for patients with PD-MCI who often have reduced mobility. Concerning gender differences, larger studies are necessary to develop personalized interventions that address specific cognitive domains needing more rehabilitation or that with different gender-related efficacy. In conclusion, our study showed that there are gender-related differences in the cognitive performances and in the cognitive stimulation efficacy in subjects with MCI-PD, with men outperforming women in global cognition and several domains, including attention and visuospatial abilities. These data suggest that gender-informed approaches may enhance the precision and impact of cognitive rehabilitative interventions in PD. Future research in neuroscience and clinical practice would benefit from integrating additional biological and psychosocial moderators, such as hormonal status, psychological resilience, and lifestyle factors, to clarify inter-individual variability in rehabilitation outcomes and further elucidate sex-related mechanisms of cognitive change in PD.

## Data Availability

The raw data supporting the conclusions of this article will be made available by the authors, without undue reservation.
