# Supplementary material for: Gender-related differences in cognitive performance and cognitive stimulation efficacy in subjects with Parkinson’s disease and mild cognitive impairment
Source: Front Aging Neurosci. 2025 Oct 22;17:1672084. doi: 10.3389/fnagi.2025.1672084 (PMC12585997; doi:10.3389/fnagi.2025.1672084)
Supplement: Supplementary file 1 [file Table_1.docx]

**Supplementary Table 1.** Mean changes (Δ T2–T1 ± SD) in men and women in the tele-rehabilitation (TR) and face-to-face (CG) groups. p-values refer to the within-group comparison (T1 vs. T2) and to the group × time interaction obtained from repeated-measures ANOVA. *Abbreviations: TR = Telerehabilitation; CG = Control Group; H-Y = Hoehn and Yahr scale; MDS-UPDRS = Movement Disorder Society-Unified Parkinson’s Disease Rating Scale; CBI = Caregiver Burden Inventory; MNA = Mini Nutritional Assessment; CIRS = Cumulative Illness Rating Scale; ADL = Activities of Daily Living; IADL = Instrumental Activities of Daily Living; MPI = Multidimensional Prognostic Index; SF-36 = 36-Item Short Form Survey.*

| **Data** | **Men** | | | | **Women** | | | |
| --- | --- | --- | --- | --- | --- | --- | --- | --- |
|  | **TR**  **Δ (T2–T1) ± DS** | **CG**  **Δ (T2–T1) ± DS** | **p-value**  **(T1 vs T2)** | **p-value**  **(Group × Time interaction)** | **TR**  **Δ (T2–T1) ± DS** | **CG**  **Δ (T2–T1) ± DS** | **p-value**  **p (T1 vs T2)** | **p-value**  **(Group × Time interaction)** |
| **MDS-UPDRS I** | 1.17 ± 4.76 | -2.31 ± 4.77 | 0.556 | 0.082 | -0.22 ± 5.38 | -0.20 ± 1.30 | 0.868 | 0.993 |
| **MDS-UPDRS II** | 0.50 ± 3.53 | -0.15 ± 3.60 | 0.811 | 0.651 | 2.33 ± 5.68 | 1.40 ± 1.95 | 0.186 | 0.732 |
| **MDS-UPDRS III** | -5.50 ± 15.91 | -2.43 ± 9.80 | 0.133 | 0.553 | 1.44 ± 13.78 | -1.60 ± 14.06 | 0.984 | 0.701 |
| **MDS-UPDRS IV** | -1.08 ± 3.58 | 0.23 ± 2.24 | 0.479 | 0.279 | 0.22 ± 1.48 | 0.00 ± 1.87 | 0.810 | 0.810 |
| **H-Y** | 0.00 ± 0.00 | 0.00 ± 0.00 | 0.775 | 0.770 | 0.33 ± 0.50 | 0.00 ± 0.00 | 0.169 | 0.169 |
| **CBI** | 0.23 ±10.90 | -4.42 ± 10.26 | 0.334 | 0.285 | -0.50 ± 4.31 | -0.325 ± 4.65 | 0.195 | 0.333 |
| **Barthel** | 0.00 ± 0.00 | 0.14 ± 0.53 | 0.365 | 0.365 | 0.00 ± 0.00 | 0.00 ± 0.00 | 0.123 | 0.123 |
| **MNA** | -0.08 ± 1.68 | 0.93 ± 2.20 | 0.288 | 0.206 | 1.78 ± 2.73 | -0.20 ± 0.45 | 0.231 | 0.140 |
| **CIRS** | 0.08 ± 0.29 | 0.21 ± 0.80 | 0.235 | 0.597 | -0.33 ± 1.00 | 0.00 ± 0.00 | 0.478 | 0.478 |
| **ADL** | 0.00 ± 0.00 | 0.00 ± 0.00 | 0.914 | 0.169 | 0.00 ± 0.00 | 0.00 ± 0.00 | 0.478 | 0.478 |
| **IADL** | 0.25 ± 0.62 | -0.07 ± 1.07 | 0.616 | 0.370 | -0.11 ± 0.33 | 0.20 ± 0.45 | 0.679 | 0.163 |
| **MPI** | 0.01 ± 0.04 | 0.00 ± 0.05 | 0.430 | 0.751 | -0.04 ± 0.05 | -0.04 ± 0.08 | 0.055 | 0.990 |
| **SF36 tot** | -31.42 ± 416.73 | -85.64 ± 451.25 | 0.501 | 0.754 | 169.67 ± 234.38 | 183.00 ± 391.16 | 0.054 | 0.937 |
